# Adverse and traumatic exposures, posttraumatic stress disorder, telomere length, and hair cortisol – Exploring associations in a high-risk sample of young adult residential care leavers

**DOI:** 10.1016/j.bbih.2022.100524

**Published:** 2022-09-30

**Authors:** David Bürgin, Vera Clemens, Nimmy Varghese, Anne Eckert, Mara Huber, Evelyne Bruttin, Cyril Boonmann, Eva Unternährer, Aoife O'Donovan, Marc Schmid

**Affiliations:** a Research Department for Child and Adolescent Psychiatry, University Psychiatric Hospitals Basel, University of Basel, Basel, Switzerland; b Department for Child and Adolescent Psychiatry and Psychotherapy, University Hospital Ulm, Ulm, Germany; c Neurobiological Laboratory for Brain Aging and Mental Health, Transfaculty Research Platform, University of Basel, Basel, Switzerland; d Department of Psychiatry and Weill Institute for Neurosciences, University of California San Francisco, San Francisco, USA; e San Francisco Veterans Affairs Medical Center, San Francisco, CA, USA

**Keywords:** Early life stress, Childhood maltreatment, Childhood trauma, Life events, Stress-biomarkers, Institutionalized children, Aging, Senescence, HPA-Axis

## Abstract

**Background:**

Childhood adversities (CAs), potentially traumatic exposures (PTEs), and posttraumatic stress disorder (PTSD) are known to increase the risk for poor health outcomes, including diseases of aging and early mortality. Telomere length (TL) and hair cortisol concentrations (HCC) are biomarkers known to be associated with CA and PTEs, and PTSD, but there is considerable heterogeneity in findings.

**Objectives:**

This study aims to investigate the association of CAs, PTEs, and PTSD with TL and HCC in a high-risk sample of young adults who were previously placed in youth residential care institutions throughout Switzerland.

**Method:**

Our sample includes 130 participants (30.8% women, M _Age_ = 26.5 ± 3.7 years) with previous youth residential care placements (M_Placements_= 3.9). CAs and PTEs, as well as PTSD, were assessed with self-reported questionnaires and semi-structured clinical interviews. Immune cell TL was measured with quantitative polymerase chain reaction (qPCR) in whole blood. Hair samples were collected for HCC measurement and assayed with high-sensitivity ELISA. Multivariate regression models were fitted to describe the associations between CAs, PTEs, and PTSD with TL and HCC, adjusting for covariates.

**Results:**

In our high-risk sample, a higher burden of CAs, PTEs, Criterion A trauma, and PTSD was associated with longer TL. PTEs, Criterion A trauma, and PTSD were associated with lower HCC, however no significant associations between CAs and HCC were found. The magnitude of these effects varied depending on the dimensional or categorical nature of the stress-phenotype and the specific measure used.

**Conclusions:**

Our findings are in contrast with many, but not all, previous studies of associations between adversity and both TL and HCC. For instance, our findings are in line with other studies that find a state of hypocortisolism in PTSD. Better measurement of adversities and trauma, multisystem biomarker approaches, and more research in larger high-risk samples at the upper end of the adversity-continuum is warranted.

## Highlights

• In this high-risk sample,childhood adversities, potential traumatic exposures, criterion A trauma, or posttraumatic stress disorder (PTSD) was associated with longer telomere length (TL).
• Potentially traumatic exposures, criterion A trauma, and PTSD were associated with lower hair cortisol concentrations.
• The magnitude of these effects varied depending on the dimensional or categorical nature of the stress-phenotype and the specific measure used.
• Hypocortisolism might explain findings of longer TL in participants with cumulated adverse and traumatic exposures.
• Research in high-risk populations is strongly needed as results across the entire spectrum of adversity exposures may not generalize to the top end of the spectrum.

## 1 Introduction

Childhood adversities (CAs) and potentially traumatic exposures (PTEs) are highly prevalent in the general population and in particular in young people who were placed out of home (Bellis et al., 2019; Fischer et al., 2016; Garcia et al., 2017; Greeson et al., 2011; Kessler et al., 2017; Kolko et al., 2010). CAs and PTEs and the accumulation of these are well known to increase the risk for long-term adverse health outcomes. They are a prerequisite for diagnoses of posttraumatic stress disorder (PTSD) and related to poor mental and physical health outcomes across the life-course including diseases of aging and metabolic disorders (Bellis et al., 2019; Clemens et al., 2018; Kessler et al., 2017; Riedl et al., 2019). Telomere length (TL) and hair cortisol concentrations (HCCs) are biomarkers known to be associated with CAs and PTEs, however the overall body of research shows considerable heterogeneity in findings, potentially due to diversity of samples, different distinct assessments of CAs and PTEs, and due to different methods of measuring cortisol and TL (Bürgin et al., 2019; Khoury et al., 2019; Li et al., 2017; Pepper et al., 2018; Ridout et al., 2017). Thus, this study aims to investigate the association between different measures of CAs, PTEs, and PTSD with TL and HCC in a highly strained at-risk sample of young adults with previous youth residential care placements.

CAs comprise a broad array of potentially harmful experiences children can make, currently defined as exposures that reflect a deviation (either absence or presence) from the expected environment that require a significant adaptation of an average child (Humphreys and Zeanah, 2015; McLaughlin, 2016; McLaughlin et al., 2014; Sheridan and McLaughlin, 2014). Such experiences of an absence of expected positive input include different forms of emotional and physical neglect and deprivation, the presence of unexpected negative inputs includes different types of abuse, violence, and trauma (Humphreys and Zeanah, 2015; McLaughlin, 2016; McLaughlin et al., 2014; Sheridan and McLaughlin, 2014). These kinds of adverse experiences are highly prevalent across different populations and are often co-occurring (Bellis et al., 2019; Bürgin et al., 2020; Copeland et al., 2007; Felitti et al., 1998; Green et al., 2010; Kessler et al., 2017; Witt et al., 2017). Next to CAs, lifetime prevalence rates of PTEs are even higher (Benjet et al., 2016; Breslau et al., 1998; Bürgin et al., 2021; Kilpatrick et al., 2013). Many studies have shown CAs and PTEs to be associated with poor health outcomes, for instance major and chronic diseases, diseases of aging, and early mortality (Baldwin and Danese, 2019; Bellis et al., 2019; Clemens et al., 2021; Gilbert et al., 2015; Johnson et al., 2020; O'Donovan et al., 2015). Thus, the reduction or elimination of CAs and PTEs should be at the center of preventive public health efforts.

Certain subpopulations within the general population are at an increased risk to have been exposed to multiple forms of CAs and PTEs as for instance out-of-home placed children and adolescents living in youth residential care or in foster families (Fischer et al., 2016; Garcia et al., 2017; Greeson et al., 2011; Jaritz et al., 2008; Kolko et al., 2010; Woods et al., 2013). Next to and resulting from such experiences, young people placed out-of-home have higher rates of mental disorders while at the same time often lacking important resources (e.g., stable family home and support networks, financial security, etc.) that other young people of their age might draw upon (Kind et al., submitted; Schmid et al., 2022b; Seker et al., 2021) – thus, they are at higher-risk for adverse long-term health as well as functional outcomes and for poor social participation and can be considered an at-risk or high-risk population at the upper end of the adversity and stress continuum compared to the general population (Schmid et al., 2022). Public health efforts to prevent and counteract adversities need more research in such “high-risk” populations to better understand which mechanisms confer risk and resilience for both mental and physical health problems.

Accelerated aging processes, as indexed by shortened telomeres, have been proposed as a mechanism linking adversities with poor health outcomes (Blackburn et al., 2015; Epel and Lithgow, 2014; Shalev, 2012). Telomeres are repeated and non-coding deoxyribonucleic acid (DNA) sequences that cap the tips of chromosomes and protect the DNA (Blackburn et al., 2015). Telomeres shorten during cell division, caused by an incomplete transcription of the chromosomes’ terminal bases; thus, telomeres shorten over repeated replication, making them an index of cellular age (Blackburn, 2005; Takubo et al., 2010). Multiple reviews and meta-analyses report overall negative associations of stress and adversity with TL. Aggregated effect sizes in these studies range from small to small-to-medium and a large heterogeneity between studies has been observed (Bürgin et al., 2019; Epel and Prather, 2018; Hanssen et al., 2017; Li et al., 2017; Mathur et al., 2016; Pepper et al., 2018; Ridout et al., 2017). In moderator analyses, this heterogeneity is in part attributed to the complexity of the adversity construct, the developmental timing of adversities and comorbidities with mental disorders, the heterogenous features of childhood trauma, and categorical versus continuous measures of stressors (Hanssen et al., 2017; Li et al., 2017; Ridout et al., 2017). Critically reviewing the meta-analytic evidence also shows that publication bias in this broader literature is likely to occur, with fill and trim analyses reducing already small effect sizes even further (Li et al., 2017; Malouff and Schutte, 2017; Pepper et al., 2018; Ridout et al., 2017). Beyond the impact of CAs on TL, TL is increasingly and extensively studied in the context of psychiatric disorders, however only a few studies to date investigated TL in individuals with PTSD, mostly in veteran samples, with mixed results (Darrow et al., 2016; Epel and Prather, 2018; Kang et al., 2020; Küffer et al., 2016; Li et al., 2017; Malouff and Schutte, 2017; Womersley et al., 2022). Taken together, a growing body of research describes associations of stress, adversity, and trauma measures, as well as mental disorders with TL – however heterogeneity is apparent in this broader literature.

Another important pathway between adversity and ill-health is the hypothalamic–pituitary–adrenocortical (HPA) axis (Kamin and Kertes, 2017; Koss and Gunnar, 2018; Zänkert et al., 2019). The HPA-axis is activated by acute stressors leading to the release of corticotropin-releasing hormone (CRH) in the hypothalamus, and the subsequent release of adrenocorticotropic hormone (ACTH) in the anterior pituitary followed by the synthesis and release of cortisol in the adrenals (Brotman et al., 2007; Bürgin, 2021; Kamin and Kertes, 2017). In the long run, cortisol down-regulates its response via negative feedback regulations (Sapolsky et al., 2000). Chronic HPA-axis activation was thus shown to lead to a chronically altered cortisol secretion, contributing to adverse health consequences (Doom and Gunnar, 2013). Numerous studies have pointed towards a significant effect of CAs on HPA axis functioning (Bruce et al., 2009; De Bellis et al., 1994; Harkness et al., 2011; Heim et al., 2000; Khoury et al., 2019), which is still evident in adulthood (Carpenter et al., 2007; Heim et al., 2000; van der Vegt et al., 2009), however, the direction of this association is not always consistent. Often cortisol was and is measured in saliva and blood, but also in urine, allowing the assessment of basal levels, daily profiles, or reactivity to acute psychological stressors and pharmacological challenges (Jiang et al., 2019). More recently, hair cortisol concentration (HCC) has emerged as a measure of systemic retrospective cortisol secretion over time, which was previously almost impossible to assess in other specimens (Dettenborn et al., 2012; Kirschbaum et al., 2009; Russell et al., 2012; Stalder and Kirschbaum, 2012; Stalder et al., 2017; Staufenbiel et al., 2013). Associated with adversities and stressors, some studies show increased hair cortisol levels and reactivity – hypercortisolism – other studies report decreased cortisol levels and reactivity as consequence of adverse exposures – thus hypocortisolism (Khoury et al., 2019; Koss and Gunnar, 2018; Stalder et al., 2017). Studies specifically focusing on chronic adversities in childhood point towards a state of hypocortisolism in those exposed to chronic adversities, which however might be spurred by psychopathology and recent and imminent stress exposures (Koss and Gunnar, 2018). Meta-analytic evidence of PTSD and cortisol points towards a state of hypocortisolism in patients with PTSD compared to healthy controls, however inconsistencies and mixed results exist depending on different approaches of measuring cortisol (Pan et al., 2018; Schumacher et al., 2019; Speer et al., 2019). Findings regarding hair cortisol are fewer compared to studies using measures from saliva, blood, and urine samples, but also trend towards a state of hypocortisolism in PTSD patients (Stalder et al., 2017; Steudte-Schmiedgen et al., 2016). Taken together, hair cortisol levels have been shown to be associated with early and chronic adversity and PTSD – however mixed findings exist.

Despite the recent interest in adversity, trauma, and their associations with accelerating aging as indexed by TL and HPA-dysregulation seen in HCC levels, there has been considerable heterogeneity in findings shown in recent reviews and meta-analyses (Bürgin et al., 2019; Khoury et al., 2019; Pepper et al., 2018). Moreover, there is a lack of studies investigating the psychophysiological sequelae of adversities and potentially traumatic exposures in high-risk populations including young people who were placed within youth residential care. This is concerning as such populations are not only underrepresented in research but overrepresented regarding their burden of mental health problems and disordersm but also physical diseases and those often exposed to chronic adversities and complex mental health burdens (Briggs et al., 2014; Hughes and Tucker, 2018; McEwen and McEwen, 2017; Zelechoski et al., 2013). Therefore, this study aims to investigate the association between CAs, PTEs, and PTSD with TL and HCCs in a high-risk population of young adults who were previously placed within youth residential care institutions in Switzerland.

## 2 Methods

### 2.1 Participants and study procedures

Participants for our cross-sectional study, were recruited into the study “Swiss Study for Clarification and Goal-Attainment in Child Welfare and Juvenile-Justice Institutions” (German: Modellversuch Abklärung und Zielerreichung in stationären Massnahmen”; MAZ.) when being placed in one of the participating Swiss youth residential care institutions between 2007 and 2012 (for an overview see Barra et al., 2022; Jäggi et al., 2021; Schmid et al., 2013). The MAZ. study had the primary aim of describing mental health and offending behavior of children and adolescents in child welfare and residential care and juvenile justice institutions. The included institutions were all accredited by the Swiss Federal Ministry of Justice represent the various types of Swiss youth institutions in regard of size, schooling opportunities, treatment options, and residing of children and adolescents (Barra et al., 2022; Jäggi et al., 2021). Participants were placed due to either civil law, penal law, or voluntarily. Participants with non-sufficient language skills and intelligence impairments had to be excluded from the MAZ.-study.

Participants were then reassessed as part of the longitudinal follow-up “Youth Welfare Trajectories: Learning from Experience” (German: "Jungenhilfeverläufe: Aus Erfahrung Lernen"; JAEL) between 2018 and 2020. Participants first, filled out online questionnaires and then came in for qualitative and structured clinical interviews. This study aimed to investigate the psychosocial health of young adults with previous residential care placements (d’Huart et al., 2022; Kind et al., submitted; Schmid et al., 2022; Seker et al., 2022). In an add-on to this study, biological specimens (blood and hair samples) were collected for biomarker assays. Participants with a common cold/flue or known immunological diseases (e.g. HIV) were excluded from bio-sampling. All participants signed an informed consent for participation in the longitudinal follow-up study and additionally for the biomarker add-on. An overview of participants run through the overall study is provided in the flow-chart in the Supplementary Material (Fig. S1). The Ethics Commission of Northwestern Switzerland (EKNZ, Ref. 2017-00718) reviewed and approved the follow-up study JAEL including its biomarker add-on.

### 2.2 Measures of childhood adversities, potentially traumatic exposures, and posttraumatic stress disorder

CAs were measured with the “Childhood Trauma Questionnaire” (CTQ) (Bernstein et al., 2003) and the “Maltreatment and Abuse Chronology of Exposure Questionnaire” (MACE-X) (Teicher and Parigger, 2015). For both these measures the dimensional severity score was used for analysis. PTEs were assessed with the “Life Events Checklist revised” (LEC-R) (Gray et al., 2004), all events that were self-experienced or witnessed were summed up to build an overall index score of PTEs. A DSM-5 Criterion A trauma, defined as being exposed to: death, threatened death, actual or threatened serious injury, or actual or threatened sexual violence, in the following way(s): direct exposure, witnessing the trauma, learning that a relative or close friend was exposed to a trauma, indirect exposure to aversive details of the trauma, usually in the course of professional duties (e.g., first responders, medics), as well as lifetime PTSD diagnoses were assessed using the “Structured Clinical Interview for DSM-5-Disorders- Clinical Version” (SCID5-CV) (First and Williams, 2016). From these interviews, we have created a categorical variable with three groups: No Criterion A trauma & no PTSD; exposed to a Criterion A trauma & no lifetime PTSD, and last exposed to Criterion A Trauma & lifetime PTSD. More detailed information regarding these measures is provided in the supplementary material.

### 2.3 Telomere length measurement

Whole blood samples were collected during the morning between 8AM and 11AM and subsequently stored at −80 °C until further use. For the LTL assay, DNA from whole blood was isolated according to the manufacturer's protocol using the FlexiGene® DNA KIT (250) (Qiagen, DE). Leukocyte TL was then measured by quantitative polymerase chain reaction (qPCR) according to methods described previously (Axelrad et al., 2013; Cawthon, 2002; O'Callaghan and Fenech, 2011). To determine TL, the T/S ratio (telomere repeat copy number [TELO] to single-copy gene number [SCG]) was calculated for each sample. The qPCR was performed using the Step One Plus system (Applied Biosystems, USA). All DNA samples were run in triplicate per plate and were assayed twice. The intra-assay CV was <1%, the inter-assay CV was 4.9%. The Ct values were analyzed with the comparative Ct method (2- ΔΔCt) relative to an internal control to be represented as the T/S ratio. As internal control, a mixture of all DNA samples was used. More detailed descriptions of the DNA extraction and qPCR procedures are found within the supplement.

### 2.4 Hair Cortisol Concentrations

Hair strains were collected from the posterior vertex region of the scalp. Due to variability on lengths of strands of hair only strands of hair of 2 cm adjacent to the scalp were analyzed reflecting the cortisol secretion over approximately the last eight weeks. Hair cortisol was extracted in line with the protocol by Gao et al. (2013). HCC was determined using a commercially available high-sensitivity (analytical sensitivity 0.007 μg/dL) cortisol enzyme immunoassay kit (Salimetrics Europe, UK) according to the manufacturer's protocols. Evaporated samples were resuspended in assay diluent provided by the manufacturer. The intra-assay coefficient of variation (CV) was 2.3%, the inter-assay CV was 6.1%. Samples were analyzed in duplicate, and mean values of respective concentrations were calculated in pg/mg hair.

### 2.5 Covariates

Internalizing mental health problem were assessed with the Youth Self-Report (YSR; Achenbach, 1991) and the Young Adult Self-Report (YASR; Achenbach, 1997) from the Achenbach System of Empirically Based Assessment (ASEBA) and used to control for the potential confounding of current internalizing psychopathology on the association between CAs and PTEs with TL and HCC*.* We used the internalizing symptoms score and transformed raw scores into T-scores. All other variables (age; sex [0 = men,1 = women], migration background of family [0 = absent, 1 = present], health status [healthy = 0, any chronic disease/illness = 1]) were assessed using self-developed anamnestic questionnaires. Migration background was categorized dichotomously as absent or present in case the participant or one parent was born outside of Switzerland and was used as a proxy for race which we do not routinely assess. Health status was screened with an item asking for “any acute or chronic disease”, as a broad index and proxy of health, participants with a common cold/flue or diseases of the immune system were excluded, based on their answer.

### 2.6 Statistical analysis

All participants with valid TL assay (N = 130) were included in the analyses. Missing data for statistical modelling was imputed using multivariate imputation by chained equation (k = 10 imputations) with its implementation in the “MICE” package using predictive mean matching (PMM) (Van Buuren and Groothuis-Oudshoorn, 2011), please find descriptives on the number of missings within the result section. After imputation, we ran multiple sets of linear regression models to predict TL and HCC from each of the four adversity and trauma measures separately (CTQ, MACE, LEC-R, and Criterion A trauma/PTSD diagnoses), adjusting for covariates (5 covariates: age, sex, migration background, health status, and internalizing psychopathology) on merged datasets. To model TL and HCC, the categorical Criterion A trauma/PTSD variable was split in two dichotomic dummy variables.

The statistical software used was R (Version 4.0.4) through RStudio (Version April 1, 1106, Boston, MA, USA). Correlation analyses and model performance were analyzed using the “easystats” ecosystem for R (Lüdecke et al., 2020, 2021; Makowski et al., 2020). Plots were created using the “ggplot2”, the “sjPlot”, and the “ggredict” packages. Our analyses approach is exploratory in nature; thus, we did not control for multiple testing. Exact p-values for all tests are reported in the tables, however we advise readers to refer to the size of standardized estimates and their corresponding 95%-confidence intervals.

## 3 Results

### 3.1 Sociodemographic characteristics

A total of 130 participants provided whole blood samples for leukocyte TL assays (30.8% women) and 92 provided hair samples for HCC analysis (38% women). The mean age of participants with valid TL data was 26.5 years (SD = 3.5, Range [16.1; 38.6], 90% between [20.0; 31.7]). Participants had a mean of 3.9 out-of-home placements (SD = 3.9). Overall, 58.5% of participants reported a migration background and 24.6% reported to have a (chronic) health condition. All participants were placed in a residential care or juvenile justice institution at baseline, 40.7% reported to have additionally been placed in a foster family. When originally included into our cohort study, 52% of participants were placed due to civil law, 24.4% were placed to penal law, and 23.6% of the participants were placed due to other reasons. Participants had a mean age of 11.4 years (SD = 4.9) when being placed for the first time and a mean age of 18.8 years (SD = 3.2) when their last placement ended. The mean duration between start of the first and end of the last placement was 7.1 years (SD = 5.2). In regard of participants treatment history, 78.5% of participants reported to have been in some sort of treatment with a psychologist or psychiatrist, 35.5% self-reported to have been diagnosed with a mental disorder, and 33.9% reported to have been in inpatient treatment in a psychiatric hospital.

Retrospective self-reports of CAs showed that 77.5% of participants screened positive for childhood adversity using the CTQ, and 90.0% were above the cut-off on at least one of the scales on the MACE. Participants reported a mean of 4.5 lifetime PTEs on the LEC-R (SD = 2.8; Range [0–12], 7.0% no PTE). In clinical interviews, 62.3% of participants reported exposure to a Criterion A traumatic event, 22.3% of the whole sample were diagnosed with lifetime PTSD (4.6% current PTSD, 17.7% past PTSD). Within the supplementary material, additional descriptive information on biomarker data, the distributions of the four adversity and trauma measures, and a correlation matrix and graphical network of all study variables of interest, and a flow-chart of participants run through the overall cohort study are displayed (Supplementary Table 2 and Supplementary Figure 1, 3-5).

For subsequent telomere models, 6.2% (N = 6) of participants had missing data in at least one variable, overall, 0.6% of data points were missing. TL data was positively skewed therefore log-transformation and standardization were performed. For HCC models, 32.3% of participants had missing data in at least one variable (N_Missing_ = 42, 36 of which due to missing hair samples), overall, 3.5% of data points were missing, the pattern of missingness is displayed in the supplement (Supplementary Fig. 2). As described in the method section missing data were multiply imputed by predictive mean matching.

### 3.2 Associations of CAs, PTEs, and PTSD with telomere length

**Fig. 1:**
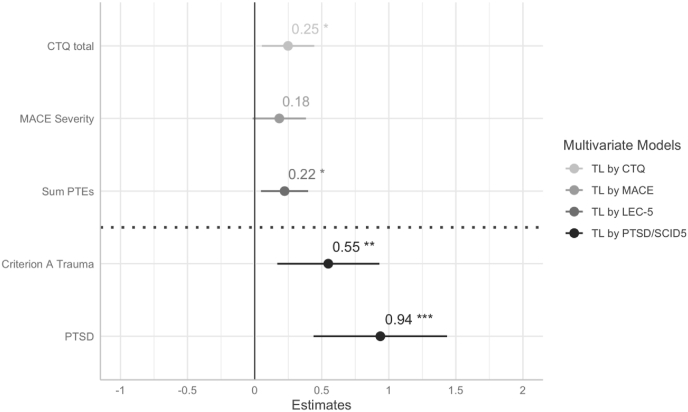
Association between adversity and trauma exposure and telomere length. Standardized beta coefficients are derived from four different multivariate linear regression models, each model was adjusted for age, sex, migration background, general health status, and internalizing mental health problems. Criterion A trauma and PTSD were compared to the reference group no Criterion A Trauma & No PTSD (below dashed line). Error bars are 95% Confidence Intervals. Full model information is found in Table 1. Significance levels are indexed at * p < .05, **p < .01, ***p < .001. CTQ=Childhood Trauma Questionnaire; MACE = Maltreatment and Abuse Chronology of Exposure Questionnaire, PTEs = Potentially Traumatic Exposures, PTSD=Posttraumatic-Stress-Disorder.

**Fig. 2:**
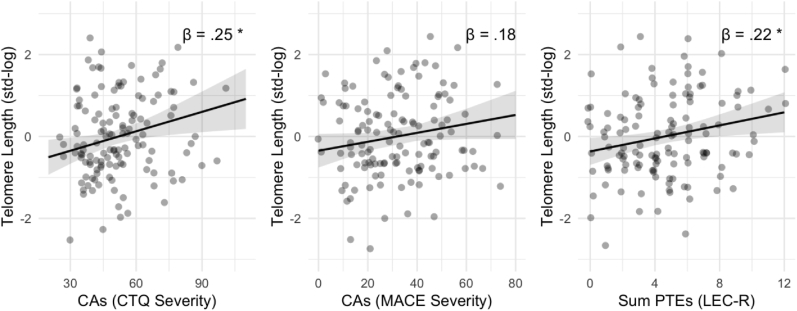
Conditional effects of self-reported dimensionally assessed Childhood Adversities (CAs; CTQ and MACE) and Potentially Traumatic Exposures (PTEs; LEC-R) on Telomere Length adjusted for age, sex, migration background, general health status, and internalizing mental health problems. Full model information is found in Table 1. Significance levels are indexed at * p < .05, **p < .01, ***p < .001.

**Fig. 3:**
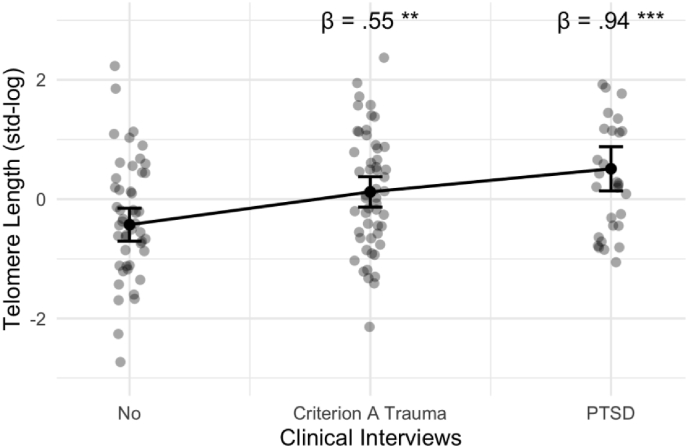
Conditional effects of categorically assessed Criterion A trauma and Lifetime PTSD diagnoses from clinical interviews and Telomere Length adjusted for age, sex, migration background, general health status, and internalizing mental health problems. Criterion A trauma and PTSD were compared to the reference group no Criterion A Trauma & No PTSD. Full model information is found in Table 1. Significance levels are indexed at * p < .05, **p < .01, ***p < .001.

In multivariate linear regression models, higher scores on the CTQ and LEC-R were significantly associated with longer TL, adjusting for age, sex, migration background, health status, and current internalizing psychopathology (see Fig. 1, Fig. 2, Table 1). The association of CAs measured with the MACE did not reach significance but trended in the same direction. Using categorical data from clinical interviews (SCID5), we found that having a Criterion A traumatic event or a diagnosis of PTSD were associated with longer TL compared to the no trauma/no PTSD group (see Fig. 1, Fig. 3, Table 1). We did not find a significance difference in TL between the trauma exposed and the PTSD group in post-hoc analyses (data not shown). Current internalizing psychopathology was not found to be significantly associated with TL in neither of the four models (see Table 1).

**Table 1:** Multivariate Regression Models on the associations of CAs, PTEs, and PTSD with TL.

	TL Model 1			TL Model 2			TL Model 3			TL Model 4		
Predictors	Estimates	CI	p	Estimates	CI	p	Estimates	CI	p	Estimates	CI	p
CTQ Total	0.25	0.05–0.44	**0.013**									
MACE Severity				0.18	−0.02–0.38	0.071						
LEC Sum Score							0.22	0.05–0.40	**0.013**			
Ref. No Exposure										**-**		
Criterion A Trauma										0.55	0.17–0.93	**0.005**
PTSD										0.94	0.44–1.43	**<0.001**
Psychopathology	−0.11	−0.31–0.08	0.256	−0.09	−0.29–0.11	0.355	−0.09	−0.28–0.10	0.336	−0.07	−0.24–0.11	0.470
Sex (Women)	−0.24	−0.64–0.16	0.236	−0.19	−0.60–0.21	0.339	−0.09	−0.47–0.29	0.632	−0.42	−0.83–−0.01	**0.043**
Age	0.13	−0.04–0.31	0.133	0.11	−0.07–0.29	0.240	0.09	−0.08–0.27	0.297	0.08	−0.09–0.26	0.340
Migration Backgr. (Yes)	−0.22	−0.58–0.14	0.220	−0.31	−0.67–0.05	0.089	−0.28	−0.64–0.07	0.116	−0.33	−0.68–0.01	0.059
Health Status (Dis.)	0.43	0.02–0.84	**0.041**	0.39	−0.02–0.81	0.063	0.37	−0.04–0.77	0.074	0.37	−0.02–0.77	0.061
Observations	130			130			130			130		
R^2^/R^2^ adjusted	0.124/0.081			0.103/0.059			0.124/0.081			0.181/0.134		

*Note.* CTQ = childhood trauma questionnaire, MACE = maltreatment and abuse chronology of exposure questionnaire, LEC = Life Events Checklist, PTSD = posttraumatic stress disorder; Psychopathology = internalizing dimensional psychopathology, TL = telomere length, CI = 95% Confidence Interval. Significance levels are indexed at * p < .05, **p < .01, ***p < .001.

### 3.3 Associations of CAs, PTEs, and PTSD with hair cortisol concentrations

**Fig. 4:**
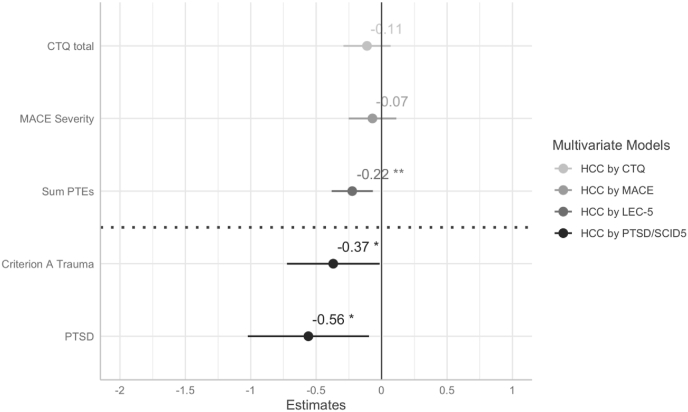
Association between adversity and trauma exposure and hair cortisol concentration (HCC). Standardized beta coefficients are derived from four different multivariate linear regression models, each model is adjusted for age, sex, migration background, general health status, and internalizing mental health problems. Criterion A trauma and PTSD were compared to the reference group no Criterion A Trauma & No PTSD (below dashed line). Error bars are 95% Confidence Intervals. Full model information is found in Table 2. Significance levels are indexed at * p < .05, **p < .01, ***p < .001. CTQ=Childhood Trauma Questionnaire; MACE = Maltreatment and Abuse Chronology of Exposure Questionnaire, PTEs = Potentially Traumatic Exposures, PTSD=Posttraumatic-Stress-Disorder.

**Fig. 5:**
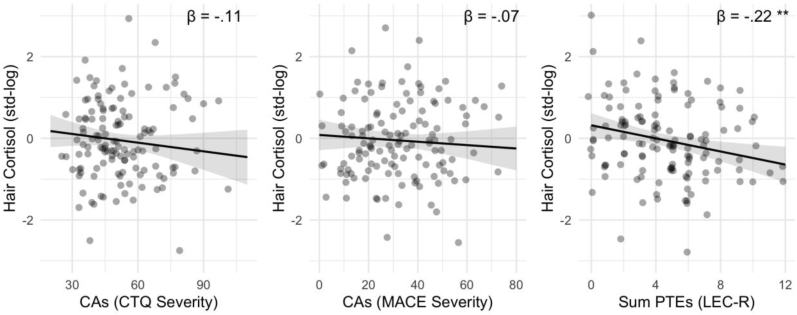
Conditional effects of self-reported dimensionally assessed Childhood Adversities (CAs; CTQ and MACE) and Potentially Traumatic Exposures (PTEs; LEC-R) on Telomere Length adjusted for age, sex, migration background, general health status, and internalizing mental health problems. Full model information is found in Table 2. Significance levels are indexed at * p < .05, **p < .01, ***p < .001.

**Fig. 6:**
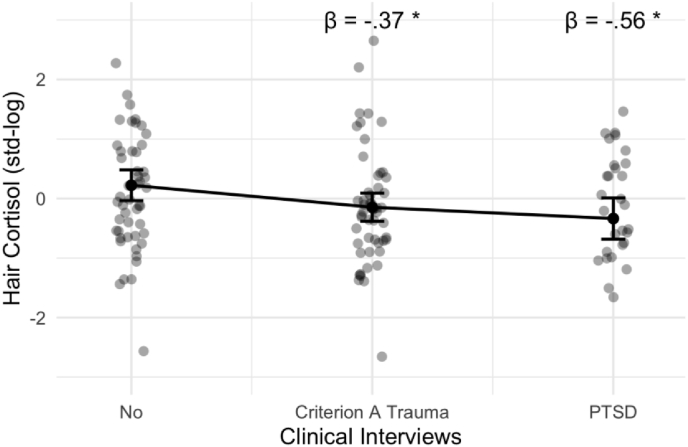
Conditional effects of categorically assessed Criterion A trauma and lifetime PTSD diagnoses from clinical interviews versus reference group without Criterion A trauma or PTSD on Hair Cortisol Concentrations adjusted for age, sex, migration background, general health status, and internalizing mental health problems. Criterion A trauma and PTSD were compared to the reference group no Criterion A Trauma & No PTSD (below dashed line). Full model information is found in Table 2. Significance levels are indexed at * p < .05, **p < .01, ***p < .001.

In multivariate linear regression models, we did not find an association between dimensionally queried CAs (CTQ and MACE) and HCC, adjusting for age, sex, migration background, health status, and current internalizing psychopathology (see Fig. 4, Fig. 5, Table 2). However, we found higher scores on the LEC-R to be associated with lower HCCs, controlling for covariates. Using categorical data from clinical interviews (SCID5), we found that having a Criterion A traumatic event or a diagnosis of PTSD was associated with lower HCCs compared to the no trauma/no PTSD group (see Fig. 4, Fig. 6, Supplementary Table 5). We did not find a significance difference in HCCs between the trauma exposed and the PTSD group in post-hoc analyses (data not shown). Additionally, current internalizing psychopathology was not found to be significantly associated with HCC in neither of the four models (see Table 2).

**Table 2:** Associations of CAs, PTEs, and PTSD with hair cortisol concentrations.

	HCCs Model 1			HCCs Model 2			HCCs Model 3			HCCs Model 4		
Predictors	Estimates	CI	p	Estimates	CI	p	Estimates	CI	p	Estimates	CI	p
CTQ Total	−0.11	−0.29–0.07	0.223									
MACE Severity				−0.07	−0.25–0.11	0.450						
LEC Sum Score							−0.22	−0.38–−0.07	0.006			
Ref. No Exposure												
Criterion A Trauma										−0.37	−0.72–−0.02	0.041
PTSD										−0.56	−1.02–−0.10	0.018
Psychopathology	−0.01	−0.19–0.16	0.872	−0.03	−0.21–0.15	0.761	0.01	−0.16–0.19	0.868	−0.03	−0.20–0.14	0.715
Sex (Women)	0.57	0.20–0.93	0.003	0.54	0.17–0.90	0.004	0.51	0.17–0.84	0.004	0.70	0.32–1.08	<0.001
Age	−0.15	−0.31–0.01	0.069	−0.14	−0.30–0.02	0.095	−0.11	−0.27–0.05	0.170	−0.12	−0.28–0.04	0.140
Migration Back. (Yes)	0.13	−0.20–0.46	0.442	0.17	−0.16–0.49	0.313	0.14	−0.17–0.46	0.369	0.18	−0.14–0.50	0.274
Health Status (Dis.)	−0.54	−0.92–−0.16	0.005	−0.52	−0.90–−0.14	0.008	−0.54	−0.90–−0.18	0.004	−0.53	−0.89–−0.16	0.005
Observations	130			130			130			130		
R^2^/R^2^ adjusted	0.154/0.113			0.148/0.106			0.196/0.157			0.189/0.142		

*Note.* CTQ = childhood trauma questionnaire, MACE = maltreatment and abuse chronology of exposure questionnaire, LEC = Life Events Checklist, PTSD = posttraumatic stress disorder; PSYCHO = internalizing dimensional psychopathology, TL = telomere length, CI = 95% Confidence Interval. Significance levels are indexed at * p < .05, **p < .01, ***p < .001.

## 4 Discussion

In this high-risk sample of young adults with previous youth residential care placements, we found that more severe burden of adversities – CAs, PTEs, or Criterion A trauma – was associated with longer age-adjusted TL. Thus, those with more CAs, lifetime PTEs, Criterion A trauma, and PTSD had longer telomeres. Regarding HCCs, we found that more severe burden of trauma exposure was significantly associated with lower HCCs (PTEs and PTSD) – findings on CAs, however, were nonsignificant. For both TL and HCC, the magnitude of the reported effects varied depending on the dimensional or categorical nature of the constructs being measured and between specific questionnaires used. Taken together, our findings confirm TL and HCC as biomarkers of CAs and PTEs, although our TL findings are in an unexpected direction within this highly strained sample at the upper end of the stress-continuum.

With regard to TL, our findings on the association of CAs with longer TL are in contrast with many studies that have indicated that more adversity is associated with shorter TL – however reviews and meta-analyses suggest aggregate effects that are rather small, heterogeneity of findings, and potential publication bias (Bürgin et al., 2019; Hanssen et al., 2017; Li et al., 2017; Pepper et al., 2018; Ridout et al., 2017). In their meta-analyses of 138 studies, Pepper et al. (2018) suggested “that the publication bias issue in this literature is not so much differential suppression of non-significant results […], but differential suppression of results that go significantly contrary to expectation.” (Suppl. Mat., p.5), underlining the importance of findings like ours. Regarding TL in PTSD, meta-analytical evidence of the first studies pointed towards shorter telomeres in patients with PTSD (Darrow et al., 2016; Li et al., 2017; Malouff and Schutte, 2017), in line with recent studies (Avetyan et al., 2019; Kim et al., 2017; Roberts et al., 2017). However, others only found shortened TL in PTSD cases with high index trauma severity (Kang et al., 2020). In contrast mixed results exist with studies reporting no differences in TL in participants with and without PTSD (Bersani et al., 2016; Solomon et al., 2017) and others reporting that PTSD is associated with longer TL cross sectionally (Küffer et al., 2016; Womersley et al., 2022) and TL lengthening longitudinally (Boks et al., 2015). The interplay between biological systems and sample characteristics may help to understand our results and are discussed. Together, our findings on CA, PTEs, and PTSD and their association with TL add to the overall complexity of this literature and contribute data from a particularly high-risk cohort of young people with previous residential care placements.

Looking at HCCs, our non-significant findings on CAs are consistent with the idea of a HPA-dysregulation; in which recent stressors are related to increased cortisol secretion whereas past stressors can be related to lower cortisol levels (Stalder et al., 2017). Studies focusing on chronic adversities in childhood indicate associations with a state of hypocortisolism, which however might be spurred by current internalizing psychopathology and recent and imminent stress exposures – underlining the need to control for the influence of current mental health when interested in the association of adversity with HCC, as done in our analytical approach (Koss and Gunnar, 2018). Our findings on HCCs and lifetime PTSD are in line with recent findings in the literature that is indicative of a state of hypocortisolism in people diagnosed with PTSD compared to healthy controls (Pan et al., 2018; Schumacher et al., 2019; Speer et al., 2019; Stalder et al., 2017; Steudte-Schmiedgen et al., 2016). Overall, however, this literature presents with mixed findings and inconsistencies exist in particular related to dose and time-dependence of effects, co-occurrence of PTSD and ongoing stressors, comorbidity with other mental disorders, and potential effects of sex (Speer et al., 2019; Steudte-Schmiedgen et al., 2016; van den Heuvel et al., 2020). Compared to basal measures of cortisol in saliva, blood, and urine samples, hair cortisol is a newer and aggregate measure of prolonged cortisol secretion and thus might be of interest for future research. Different biological systems and sample characteristics are discussed below to partly explain our results. Together, our findings complement previous research by investigating a cohort of young adults with previous residential care placements at the upper end of the stress-continuum using different measures of “stress-phenotypes” and replicating findings of hypocortisolism in this unique sample.

Our partly unexpected findings on the direction of the association between PTEs, PTSD, HCC and TL need to be discussed in regard to three important areas – (1) interconnected biological systems and pathways; (2) sample and population specifics; and (3) stress-theoretical considerations. First, we investigated two different biomarkers from different specimens – blood and hair – both markers are studied extensively. Comparable to the enormous interest in TL and HPA maintenance and functioning as separate outcomes, the literature on the interconnection of TL and cortisol is rather small (Jiang et al., 2019). The interplay of both these systems might help us to understand the rather counterintuitive finding of longer TL with more adversity. Hypocortisolism – as discussed above – seems to be apparent in people exposed to prolonged and complex CAs and PTEs and those having PTSD (Koss and Gunnar, 2018; Speer et al., 2019; Zänkert et al., 2019). Higher cortisol, in vitro and following acute stressors in humans, has been shown to reduce the activity of the enzyme telomerase (Choi et al., 2008; Epel et al., 2010), which can lengthen telomeres. In contrast, chronic stress appears to upregulate telomerase activity in animal models (Beery et al., 2012). Thus, hypocortisolism following chronic adversity and PTSD in our sample might lead to an upregulation of telomerase that could lengthen telomeres. We recently were able to show that TL and HCC are strongly associated with adjusted standardized associations of large magnitude (std. beta = .67) in data from this very same study (Bürgin et al., 2022). The original idea of telomeres as simplistic mitotic clocks has now evolved into a complex picture of transgenerational involvement and interconnected molecular and cellular pathways interacting (Lin and Epel, 2022), of which telomere maintenance and HPA-regulation are key players.

Second, some important sample-specific considerations need to be discussed to better understand our results. Young adults with previous residential care placements are a rather heterogenous group with many having a migration background and co-occurring health problems; they are a high-risk population with lower resources and are at the upper end of the stress-continuum compared to young people in the general population (Jäggi et al., 2021; Schmid et al., 2022; Seker et al., 2021). Thus, even if such samples are stratified according to a specific risk factor (e.g. exposure to CAs and PTEs), those without this specific risk factor might not be low risk but have a different risk profile (Kind et al., submitted). Furthermore, while specific selection effects are often discussed while studying older cohorts, they might also be apparent even at younger ages in high-risk populations (e.g., selective morbidity/mortality and institutionalization) (Bürgin et al., 2020; Küffer et al., 2016). Taken together, this study examines a naturalistic sample characterized by high-strain and broad comorbidities of different types of risk factors, thus our findings should be understood as adding to our understanding of the sequelae of adverse exposures at the high end of the stress and adversity spectrum.

Third, the overall body of research on long-term outcomes of adversity and stressors suffers from inconsistencies in conceptualization and measurement of adversities and stressors – with recent debate about how to move the field forward (Bürgin, 2021; Danese, 2020; Epel et al., 2018; Kagan, 2016; McLaughlin, 2016; Slavich, 2019). These inconsistencies were previously shown to explain part of the heterogeneity observed in reviews and meta-analyses on the association of adversities and stressors with TL (Bürgin et al., 2019; Hanssen et al., 2017; Li et al., 2017; Ridout et al., 2017). Considering this heterogeneity, we used different measures of adversity and trauma and found variations in effects sizes depending on these measures – with the largest effects found for categorical measures from clinical interviews. Better understanding the complex nature of adversity and stress phenotypes will help to elucidate long-term trajectories and help to develop strategies to counteract adversity-associated health risks and to foster resilience in the face of adversity.

Findings of the present study also need to be interpreted considering some important shortcomings and limitations. First, the sample size of this study (N = 130) is relatively small, thus some of the estimates have large confidence intervals and might partly be underpowered. The sample, thus, had a low prevalence of current (4.6%) and past PTSD (17.7%), thus only life-time PTSD was used in statistical models limiting the generalizability of these specific findings. Due to the small and diverse sample, associations reported here should be considered exploratory and replication is needed, especially related to our rather unexpected findings of a positive association of childhood adversity and PTEs with TL, as well as the positive trend of age and TL. Still, findings from small studies in diverse and special high-risk samples add an important piece to the larger puzzle of understanding stress-related dysregulation in those cumulating risk-factors. Second, our study is cross-sectional in nature, and we used retrospective measures of CAs and PTEs with their debated shortcomings (e.g., recall biases) (Bürgin et al., 2020; Crosswell and Lockwood, 2020; Danese, 2020; Danese and Widom, 2020; Hardt and Rutter, 2004). However, self-reports were complemented using data from clinical interviews conducted by trained psychologists, which might reduce some recall biases (Baldwin et al., 2019). Third, no control group of young people without residential care placements is apparent as well as no clinical PTSD population. Considering the small and heterogenous sample and the lack of a control group, we did not adjust models for placement characteristics such as cumulative time or number of or number of different kind of institutions. Fourth, migration background was used as proxy for race/ethnicity as this is not assessed by default within Europe, however, might co-occur with specific migration and race/ethnicity related additional stressors. Despite these shortcomings, this study investigates a rare and well phenotyped high-risk population – young adults with previous residential care placements – with standardized adversity and trauma measures, structured clinical interviews, and two biomarkers from different specimens and thus adds to our current understanding of the consequences of adversity in those more likely to be at higher risk.

## 5 Implications

Notwithstanding these limitations, there are several important implications of this study. First, research in high-risk populations, such as children in residential care and those leaving the care systems – is strongly needed as results across the entire spectrum of adversity exposure may not generalize to the top end of the spectrum. Adding psychoneuroendocrinological stress-measures to such studies or to routine care in the long run might help to better predict adverse outcomes in the long run as for instance rehospitalization in those with history of adversity or severe mental illness (Bartoli et al., 2020). Second, clearer agreed-upon conceptualization and comprehensive measurement of adversity and stressors is needed to move the field forward, this should include incorporation of non-invasive biological measures of stress such as hair cortisol to expand the stress-phenotype measured (Crosswell and Lockwood, 2020; Epel et al., 2018; McLaughlin, 2016). Third, future research might benefit from multisystem approaches investigating different important markers and systems simultaneously over time to better understand dose and time dependence of dysregulation across different systems (Entringer and Epel, 2020; Koss and Gunnar, 2018; Kuhlman et al., 2017; Reid and Danese, 2020). Also, it is important to acknowledge that the interplay of systems, processes of homeostasis and allostasis, might not per se be linear, but be more dynamic, non-linear having potential threshold effects (Bürgin et al., 2022). In particular the inflammatory (Lacey et al., 2020), oxidative stress, and redox systems (Alameda et al., 2018; Picard and McEwen, 2018) are of high promise for future research to better understand the complex interactions of systems. Taken together, we need to be challenged to raise the bar and thus the quality of research on long-term outcomes of adversity, trauma, and its sequelae addressing complex “stress-phenotypes” as predictors and interconnected systems as outcomes and to continue to challenge our own paradigms to improve care and prevention efforts to counteract risks of adversity exposed young people (Bürgin, 2021; Danese, 2020; Lin and Epel, 2022).

## 6 Conclusion

In this high-risk sample of residential care leavers, a higher burden of early adversity and trauma was associated with longer telomeres. Trauma exposure and PTSD, however, were associated with lower hair cortisol concentrations. Our findings that adversity and trauma are associated with longer TL are contrary to many studies but not all prior studies and add findings from a rare high-risk sample to the complexity of this literature. Our findings on PTEs, PTSD and HCCs are in line with other studies that find a state of hypocortisolism related to chronic adversity and PTSD. Thus, hypocortisolism might explain findings of longer TL in participants with high adversity and trauma load. Better measurement of adversities and trauma, multisystem biomarker approaches, and more research in high-risk samples is warranted.

## Author contributions

Conceptualization, DB, VC, MS, and AOD; biomarker analyses AE and NV; data analysis, DB and EU; writing—original draft preparation, DB, VC, and AOD; writing—review and editing, MH, EB, EU, VC, CB, and MS; supervision, MS and AOD.; project administration, DB, MH, EB, and CB; funding acquisition, MS, and DB; All authors have read and agreed to the published version of the manuscript.

## Funding

The overall longitudinal cohort study JAEL investigating the psychosocial burdens of young adults with previous residential care placements was funded by the Swiss 10.13039/501100003558Ministry of Justice (PI MS). The neurobiological add-on study in which blood samples were drawn and anylazed was funded by the Gertrude Thalmann Foundation of the University Psychiatric Hospitals Basel, Switzerland (PI MS and DB). AOD was supported by a US NIMH K01 Award (K01MH109871). DB was supported by two fellowships from the Dr. Botond Berde Funds of the of the “Freie Akademische Gesellschaft” Basel, Switzerland.

## Institutional review board statement

The study was conducted in accordance with the Declaration of Helsinki and approved by the Ethics Committee of Northwestern Switzerland (EKNZ; Ref. 2017-00718; 30.06.2017).

## Informed consent statement

Written informed consent was obtained from all subjects involved in the study.

## Declaration of competing interest

The authors declare no conflict of interest. The funders had no role in the design of the study; in the collection, analyses, or interpretation of data; in the writing of the manuscript, or in the decision to publish the results.

## Data Availability

Data will be made available on request.
