# Supplementary material for: Adverse and traumatic exposures, posttraumatic stress disorder, telomere length, and hair cortisol – Exploring associations in a high-risk sample of young adult residential care leavers
Source: Brain Behav Immun Health. 2022 Sep 30;26:100524. doi: 10.1016/j.bbih.2022.100524 (PMC9535425; doi:10.1016/j.bbih.2022.100524)
Supplement: Multimedia component 1 [file mmc1.docx]

**Adverse and traumatic exposures, posttraumatic stress disorder, telomere length and hair cortisol – Exploring associations in a high-risk sample of young adult residential care leavers**

David Bürgin^1,2,4,5^ *, Vera Clemens^2^ *, Nimmy Varghese^3^, Anne Eckert^3^, Mara Huber^1^, Evelyne Bruttin^1^, Cyril Boonmann^1^, Eva Unternährer^1^, Aoife O’Donovan^4,5^ * & Marc Schmid^1^ *

**Supplementary Methods**

**Childhood Trauma Questionnaire (CTQ)**

To assess childhood adversity, the “Childhood Trauma Questionnaire-Short Form” (CTQ-SF) was used as a retrospective self-report instrument (Bader et al., 2009). The CTQ and its short version CTQ-SF are the most widely used questionnaires internationally to assess experiences of abuse and neglect before the age of 18. Based on the German norm data, severity classifications can be formed ("none-minimal", "slight-moderate", "moderate-severe" to "severe-extreme") (Hauser et al., 2011; Witt et al., 2017). Participants over the cut-score of moderate-to-severe on any of the subscales were considered to have been exposed to adversity in descriptive analyses. However, we only used the total score of the CTQ as an overall indicator of the severity of childhood adversity in our regression models.

**Maltreatment and Abuse Chronology of Exposure Questionnaire (MACE)**

The MACE is a screening instrument designed to assess interpersonal maltreatment in childhood up to adolescence (till reaching the age of 18). It includes 10 subscales measured with 75 items: Verbal Violence by Parents, Nonverbal Emotional Violence by Parent, Emotional Neglect, Physical Neglect, Witnessed Physical Violence between Parents, Witnessed Violence to Siblings, Witnessed Violence to Children, Witnessed violence against siblings, Emotional violence by peers, Physical violence by peers, Sexual violence, Loss of a parent. If an experience is specified as having been lived through, the age and the period over which the abuse took place are also asked (Isele et al., 2014; Teicher & Parigger, 2015). Due to the sensitive nature of the questions and their possible stressful effects on the participants' well-being, the questionnaire was conducted during face-to-face interviews. Participants scoring above the cut-off on any of these scales were exposed to adversity on the MACE. We, however, used the MACE multiplicity score as an indicator of the severity of exposure to childhood adversities in our regression analyses.

**Life Events Checklist revised (LEC-R)**

The LEC-R is a questionnaire that captures the experience of potentially traumatizing events (PTEs) across the life-course (Gray et al., 2004). It contains a list of 19 difficult and stressful events and was implemented as a computer-based screening in the study. All events that were self-experienced or witnessed were summed up to build an overall score of PTEs. This score was then used as predictor variable within this study.

**Structured Clinical Interview for DSM5-Disorders- Clinical Version (SCID5-CV)**

The SCID-5-CV is a research-constructed, semi-structured clinical interview based on the adult DSM-5 disorders (First et al., 2015). It captures the following 10 dimensions: Affective Episodes, Psychotic and Associated Symptoms, Differential Diagnosis of Psychotic Disorders, Differential Diagnosis of Affective Disorders, Disorders Associated with Psychotropic Substances, Anxiety Disorders, Obsessive-Compulsive Disorder and Post-Traumatic Stress Disorder, Attention-Deficit/Hyperactivity Disorder, Screening for Other Disorders, Adjustment Disorder. Within this study we used the life-time diagnoses of Post-Traumatic Stress-Disorder as a predictor variable.

**Detailed information on DNA extraction**

The DNA of the cells was isolated according to the FlexiGene DNA Handbook using the FlexiGene^®^ DNA KIT (250) (Qiagen, DE). Briefly, 300 ul of whole blood was mixed into 750 μl buffer FG1 and was centrifuged at 10000 *x* g for 20 s. In the next step, 150 μl FG2/GIAGEN protease was mixed into the tube and then incubated at 65°C for 5 min. Afterwards, 150 μl of isopropanol (100 %) was added. Then, the tube was vortexed until a DNA precipitate was observed. This step was followed by a centrifugation for 5 min at 10000 *x* g. The supernatant was discarded, and the pellet was resuspended in 150 μl of 70 % ethanol by vortexing for 5 sec. After discarding the supernatant, the DNA pellet was air-dried until complete liquid evaporation. The DNA pellet was then dissolved in 200 μl buffer FG3 by vortexing for 5 sec at low speed, followed by an incubation for 60 min at 65 °C in a heating block. The DNA content was assessed by using a Nanodrop 1000 spectrophotometer (Thermo Fisher Scientific, USA). All DNA samples were stored in -80°C until further procedure.

**Telomere length determination by qPCR**

The telomere length was measured by quantitative polymerase chain reaction (qPCR) according to the method described previously (Axelrad et al., 2013; Cawthon, 2002; O'Callaghan & Fenech, 2011). To determine the telomere length, the T/S ratio (telomere repeat copy number (TELO) to single-copy gene number (SCG)) was accessed, which represents a relative measure to the telomere length. The TELO and SCG was detected for each DNA sample. Table 1 lists the forward and reverse primers of TELO and SCG (ß-globin). For the qPCR, a master mix was prepared. The composition of one reaction of TELO master mix was 1.08 ul teloF primer (54 nM), 1,6 μl teloR primer (80 nM), 5,32 μl H2O and 10 ul SYBR green master mix and that of the SCG master mix was 1,6 μl ß-globinF primer (80 nM), 1,6 μl ß-globinR primer (80 nM), 4,8 μl H2O and 10 μl SYBR green master mix. The master mixes of TELO or SCG (18 μl per well) were loaded into 96-well-plates. Afterwards, 2 μl of the DNA sample, at a final concentration of 10 ng/μl, was added to the corresponding wells. The qPCR was performed using the Step One Plus system (Applied Biosystems, USA). All DNA samples were run in triplicate and were assayed twice at two different time points. The qPCR settings were first the initial denaturation at 95°C for 10 min, followed by a cycling of 50 repeats containing 1.) 10 sec hold at 95°C and 2.) 60 sec hold at 58 °C. The Ct values were exported with the SABiosciences PCR Array Data Analysis Software and analyzed with the comparative Ct method (2^- ∆∆Ct^) relative to an internal control to be represented as the T/S ratio. As internal control, a mixture of all DNA samples was used.

**Suppl. Table 1.** Forward and reverse primer sequences of TELO and SCG.

| **Primer** | **Sequence (5’-3’)** |
| --- | --- |
| teloF | CGGTTTGTTTGGGTTTGGGTTTGGGTTTGGGTTTGGGTT |
| teloR | GGCTTGCCTTACCCTTACCCTTACCCTTACCCTTACCCT |
| ß-globinF | GCTTCTGACACAACTGTGTTCACTAGC |
| ß-globinR | CACCAACTTCATCCACGTTCACC |

Reason for drop-out (*n*=38):

- Hair too short (n=21)
- Other (n=17)

Reason for drop-out (n=55):

- Afraid of needles (n=4)
- Digital Assessments (n=8)
- Weekend Assessments/ lab not open (n=7)
- Did not want to (n=13)
- Other (n=23)

**Biomarker Sample (t2)**

Hair samples for HCCs assays (*N*=92)

Reason for drop-out (n=81):

- Did not provide informed consent for follow-up (n=81)

**Follow-Up (JAEL, t2)**

**Baseline (MAZ, t1)**

Reason for drop-out (*n*=46):

- Unable to schedule a meeting and motivate participants to come to the in-person interviews (n=46)

Whole blood samples for TL assays (*N*=130)

Reason for drop-out (n=280):

- Refused to participate (n=99)
- Oral agreement, but never participated (n=44)
- Could not be reached (n=129)
- Were deceased (n=8)

Included in the follow-up JAEL study (*N*=231)

Participants with consent for follow-up

(*N*=511)

Included in the baseline MAZ. study (*N*=592)

Face-to-Face Interviews of JAEL (*N*=185)

**Supplementary Figure 1.** Flow Chart of Study Participants through the study with final sample sizes included in analyses.

**Supplementary Information on Missing Data Patterns**

**
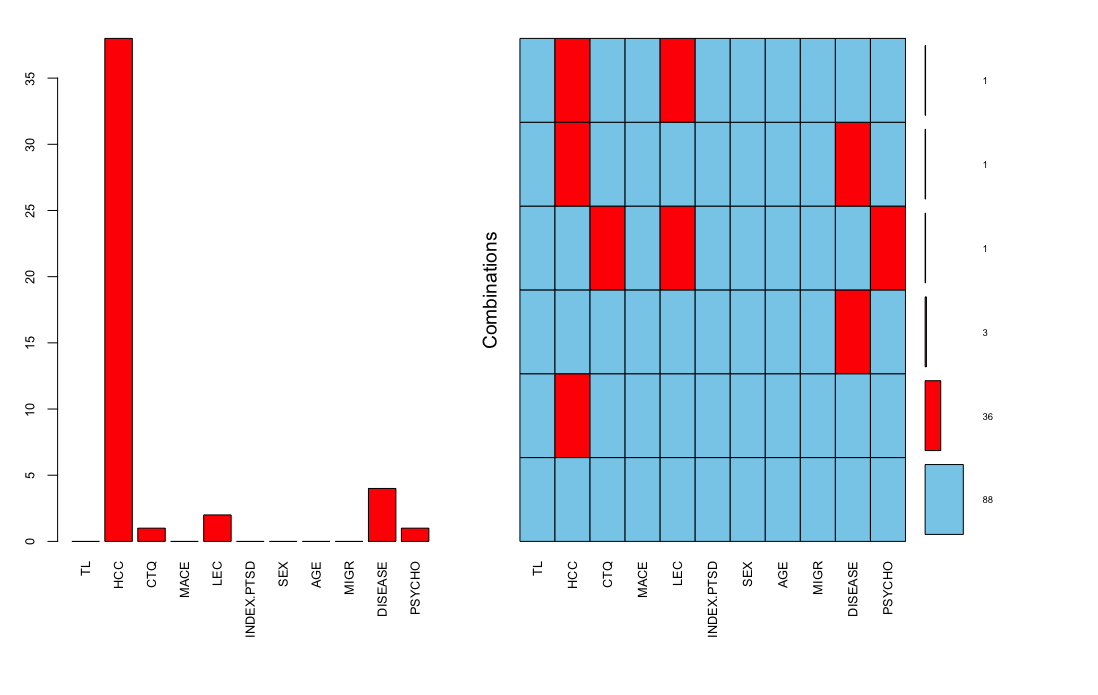
**

**Supplementary Figure 2.** Missing data pattern of all variables used in the study. TL = telomere length. HCC = hair cortisol concentration, CTQ = childhood trauma questionnaire, MACE = maltreatment and abuse chronology of exposure questionnaire, LEC = Life Events Checklist, PTSD = posttraumatic stress disorder; MIGR = Migration Background, PSYCHO = internalizing dimensional psychopathology.

**Supplementary Results**

**Supplementary Table 2.** Descriptives of biomarker data.

|  | **TL**  **(N=130)** | **z-log TL (N=130)** | **HCC (N=92)** | **z-log HCC (N=92)** |
| --- | --- | --- | --- | --- |
| Mean | 0.9 | 0 | 13.3 | 0 |
| SD | 0.3 | 1 | 11.4 | 1 |
| Median | 0.8 | -0.1 | 9.4 | -0.1 |
| Min | 0.4 | -2.7 | 1.6 | -2.6 |
| Max | 1.9 | 2.5 | 78.2 | 2.8 |
| Range | 1.6 | 5.3 | 76.6 | 5.5 |
| Skew | 0.9 | 0.1 | 2.8 | 0.2 |
| Kurtosis | 0.4 | -0.3 | 11.0 | 0.0 |

**
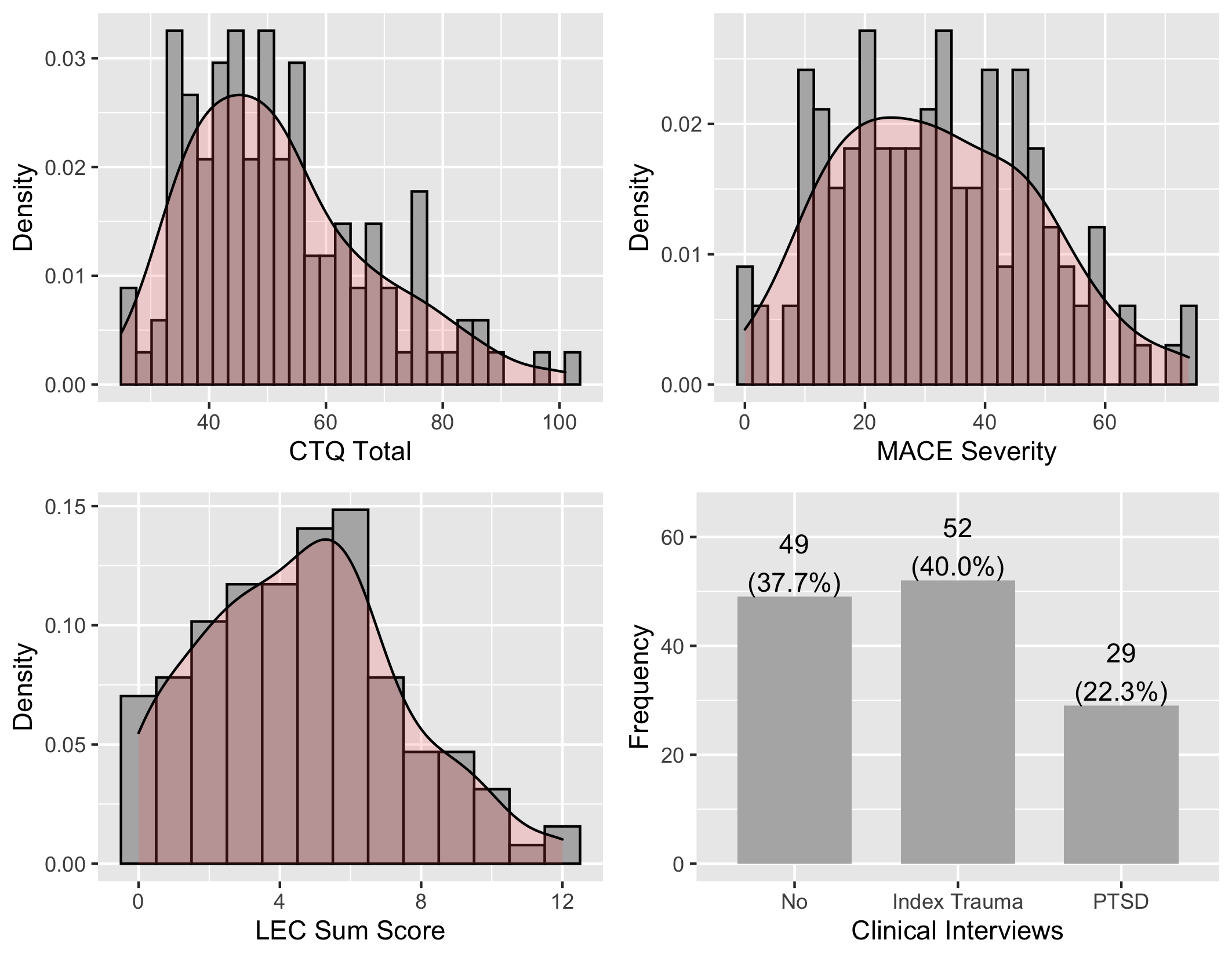
***Note.* Z-log means after using the natural logarithm and z-standardization. TL=telomere length, HCC = hair cortisol concentrations.

**Supplementary Figure 3.** Descriptives on main predictor variables used in the study. CTQ = childhood trauma questionnaire, MACE = maltreatment and abuse chronology of exposure questionnaire, LEC = Life Events Checklist, PTSD = posttraumatic stress disorder.

**
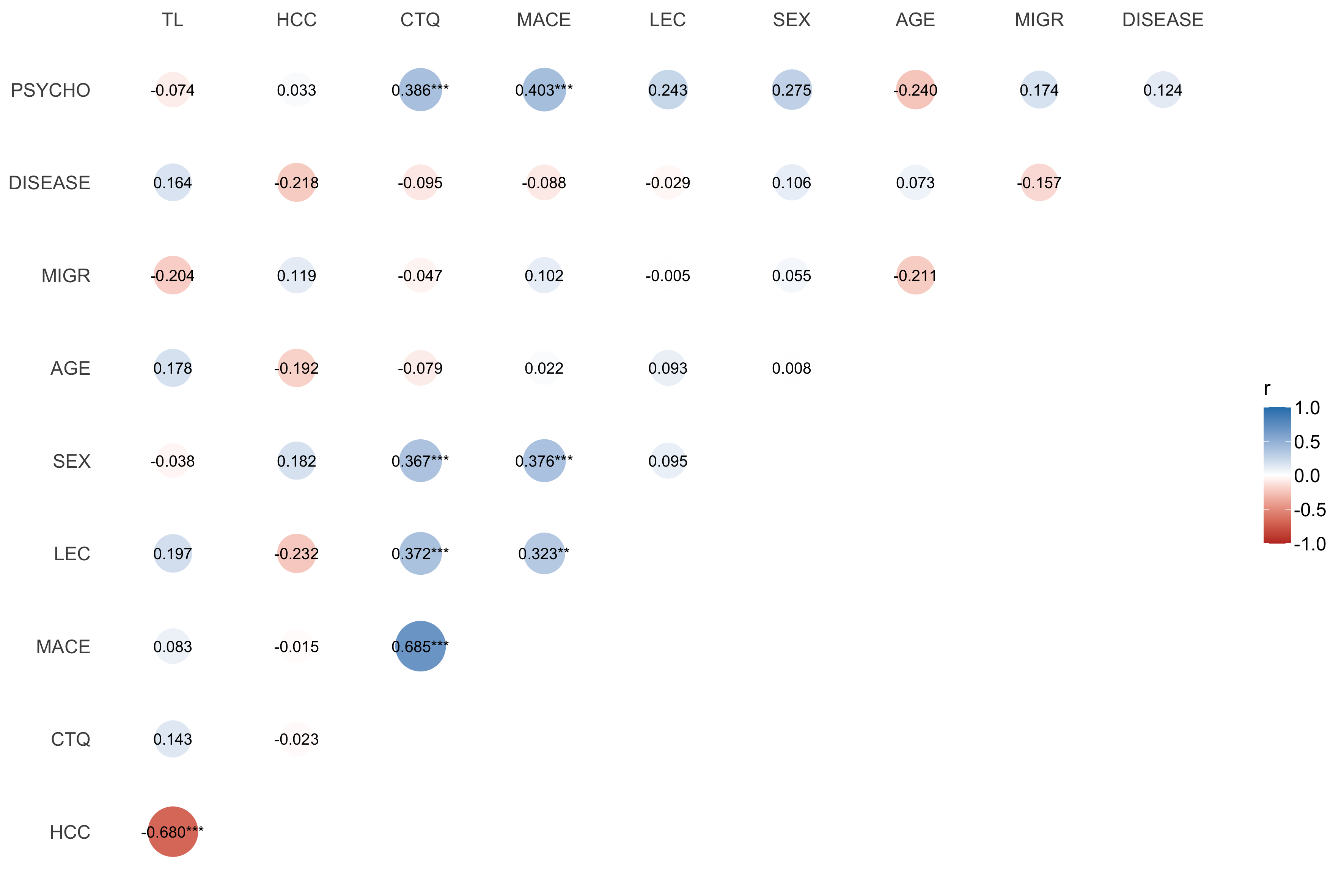
**

***Supplementary Figure 4.*** Correlation Matrix of all study variables. Correlation matrix are Pearson correlations, p-values are adjusted with the method by Holm (1979). The number of observations varies between 92-130. Plots were created with the “correlation” and the “see” package from the “easystats” ecosystem in R. TL = telomere length. HCC = hair cortisol concentration, CTQ = childhood trauma questionnaire, MACE = maltreatment and abuse chronology of exposure questionnaire, LEC = Life Events Checklist, MIGR = Migration Background, PSYCHO = internalizing dimensional psychopathology. Significance levels are indexed at * p<.05, ** p<.01, *** p<.001.


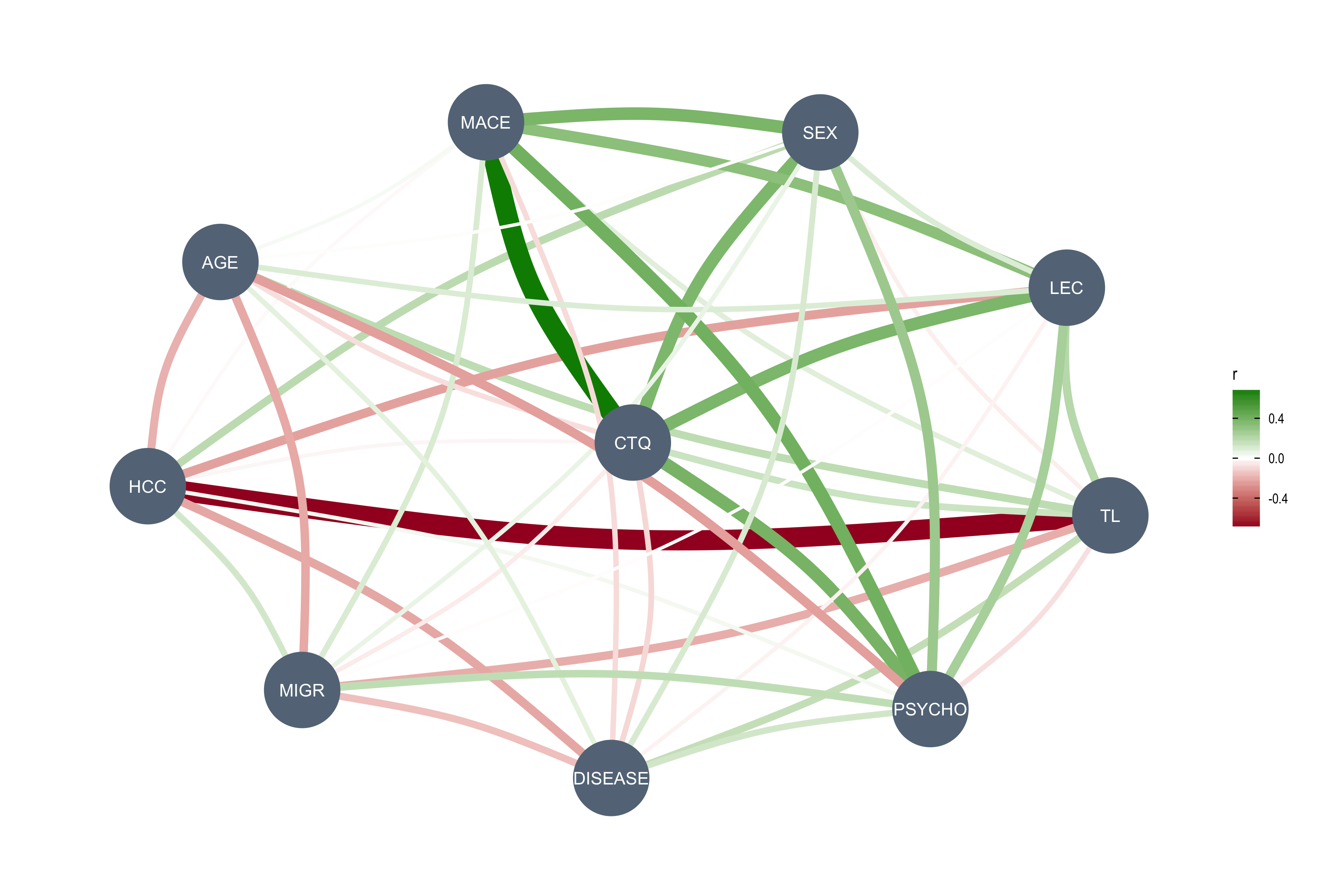


***Supplementary Figure 5****.* Gaussian Graphical Model (GGM) of all study variables and their Pearson Correlations. A GGM include a set of variables depicted as circles (“nodes”), and a set of lines that visualize relationships between them, which thickness represents the strength of association and the color the direction of association, thus it’s a graphical representation of the correlation matrix from the previous page. Plots were created with the “correlation” and the “see” package from the “easystats” ecosystem in R. TL = telomere length. HCC = hair cortisol concentration, CTQ = childhood trauma questionnaire, MACE = maltreatment and abuse chronology of exposure questionnaire, LEC = Life Events Checklist, PTSD = posttraumatic stress disorder; MIGR = Migration Background, PSYCHO = internalizing dimensional psychopathology.

**References**

Axelrad, M. D., Budagov, T., & Atzmon, G. (2013). Telomere length and telomerase activity; a Yin and Yang of cell senescence. *J Vis Exp*(75), e50246. <https://doi.org/10.3791/50246>

Bader, K., Hänny, C., Schäfer, V., Neuckel, A., & Kuhl, C. (2009). Childhood trauma questionnaire–psychometrische Eigenschaften einer deutschsprachigen Version. *Zeitschrift für Klinische Psychologie und Psychotherapie, 38*(4), 223-230.

Cawthon, R. M. (2002). Telomere measurement by quantitative PCR. *Nucleic Acids Res, 30*(10), e47. <https://doi.org/10.1093/nar/30.10.e47>

First, M., Williams, J., Karg, R., & Spitzer, R. (2015). Structured clinical interview for DSM-5 disorders, clinician version (SCID-5-CV). *Arlington, VA: American Psychiatric Association*.

Gray, M. J., Litz, B. T., Hsu, J. L., & Lombardo, T. W. (2004). Psychometric properties of the life events checklist. *Assessment, 11*(4), 330-341. <https://doi.org/10.1177/1073191104269954>

Hauser, W., Schmutzer, G., Brahler, E., & Glaesmer, H. (2011). Maltreatment in childhood and adolescence: results from a survey of a representative sample of the German population. *Dtsch Arztebl Int, 108*(17), 287-294. <https://doi.org/10.3238/arztebl.2011.0287>

Isele, D., Teicher, M. H., Ruf-Leuschner, M., Elbert, T., Kolassa, I.-T., Schury, K., & Schauer, M. (2014). KERF–ein Instrument zur umfassenden Ermittlung belastender Kindheitserfahrungen. *Zeitschrift für Klinische Psychologie und Psychotherapie*.

O'Callaghan, N. J., & Fenech, M. (2011). A quantitative PCR method for measuring absolute telomere length. *Biol Proced Online, 13*, 3. <https://doi.org/10.1186/1480-9222-13-3>

Teicher, M. H., & Parigger, A. (2015). The 'Maltreatment and Abuse Chronology of Exposure' (MACE) scale for the retrospective assessment of abuse and neglect during development. *PLoS One, 10*(2), e0117423. <https://doi.org/10.1371/journal.pone.0117423>

Witt, A., Brown, R. C., Plener, P. L., Brahler, E., & Fegert, J. M. (2017). Child maltreatment in Germany: prevalence rates in the general population. *Child Adolesc Psychiatry Ment Health, 11*(1), 47. <https://doi.org/10.1186/s13034-017-0185-0>
